# Contribution of medical senior house officers to a medical referral in the emergency department

**DOI:** 10.1186/cc14588

**Published:** 2015-03-16

**Authors:** GF Fitzpatrick

**Affiliations:** 1 University Hospital, Limerick, Ireland

## Introduction

In Irish hospitals, the medical senior house officer (SHO) is the most junior fully qualified doctor on the medical on-call team. After a patient has been seen by an emergency department doctor of any level, they are almost always referred directly to the medical SHO. This process has been shown to delay a patient's ward admission by 3 hours 30 minutes [1]. We attempted to quantify the additional benefit for the patient of being seen by the on-call medical SHO, in terms of patients discharged, new diagnoses reached, and new treatments initiated.

## Methods

The emergency department notes and clinical charts of 182 patients were assessed. This constituted a random sample of patients referred by emergency department doctors to the medical team on call over a 2-month period (November to December 2011).

## Results

Discharged: 3/182 (1.6%) of patients referred to the medical team were discharged directly by the medical SHO. Diagnosed: medical SHOs suggested a diagnosis which was different from, or additional to, the ED doctor, in 52/182 cases (28.6%). However, the medical consultant only agreed with this diagnosis in 25 cases (13.7%). This means an incorrect new diagnosis was reached more often than not (14.9%). Treatment: the majority of cases (116/182 (63.7%)) saw no new treatment initiated by the medical SHO. Of the rest, only 31 (17%) had a new treatment initiated by the medical SHO which was continued on by the medical consultant through the admission.

## Conclusion

Few direct discharges, new diagnoses, or key new treatments were initiated by the medical SHO in the emergency department. A paper from our hospital shows that more patients referred in by GPs to ED are admitted compared with those referred in to the acute medical assessment unit, with comparable disease severity (43% vs. 12.5) [2]. That paper highlighted the fact that the junior level of the medical NCHDs who see patients in the ED may contribute to their lack of discharging/decision-making zeal. Our survey further illustrated this feature. Our study provided no evidence that a formal medical assessment should delay a patient progressing to the medical ward. Additional genuine urgent OPD appointment slots could be another beneficial measure.
